# Morquio Syndrome: A Case Report

**DOI:** 10.7759/cureus.2270

**Published:** 2018-03-05

**Authors:** Kamleshun Ramphul, Stephanie G Mejias, Yogeshwaree Ramphul-Sicharam

**Affiliations:** 1 Department of Pediatrics, Shanghai Xin Hua Hospital Affiliated to Shanghai Jiao Tong University School of Medicine, Shanghai, People's Republic of China; 2 Department of Pediatrics, Robert Reid Cabral Children's Hospital Affiliated to the University Iberoamericana Unibe School of Medicine; 3 Sir Seewoosagur Ramgoolam National Hospital

**Keywords:** morquio syndrome, mucopolysaccharidosis

## Abstract

Mucopolysaccharidosis type IV (MPS IV), also known as Morquio syndrome, is a rare autosomal recessive lysosomal storage disease. The main features include skeletal defects and possible cardiopulmonary complications. The cost of diagnosing and treating this condition is high, and treatment is not easily available everywhere. We present a case of Morquio syndrome seen in a seven-year-old male from Iraq with multiple skeletal deformities.

## Introduction

Mucopolysaccharidosis type IV (MPS IV), also known as Morquio syndrome, is a rare autosomal recessive lysosomal storage disease. The incidence of this disease ranges between 0.14 to 0.22 per 100,000 births [1]. Its main features include skeletal disorders such as short-trunk dwarfism, dental abnormalities, and possible cardiopulmonary complications. Morquio syndrome consists of MPS IV A, which results from mutations in galactosamine-6-sulfatase genes, and MPS IV B, which is due to beta-galactosidase deficiency [2-3]. We present a case of Morquio syndrome observed in Iraq.

## Case presentation

**Figure 1:**
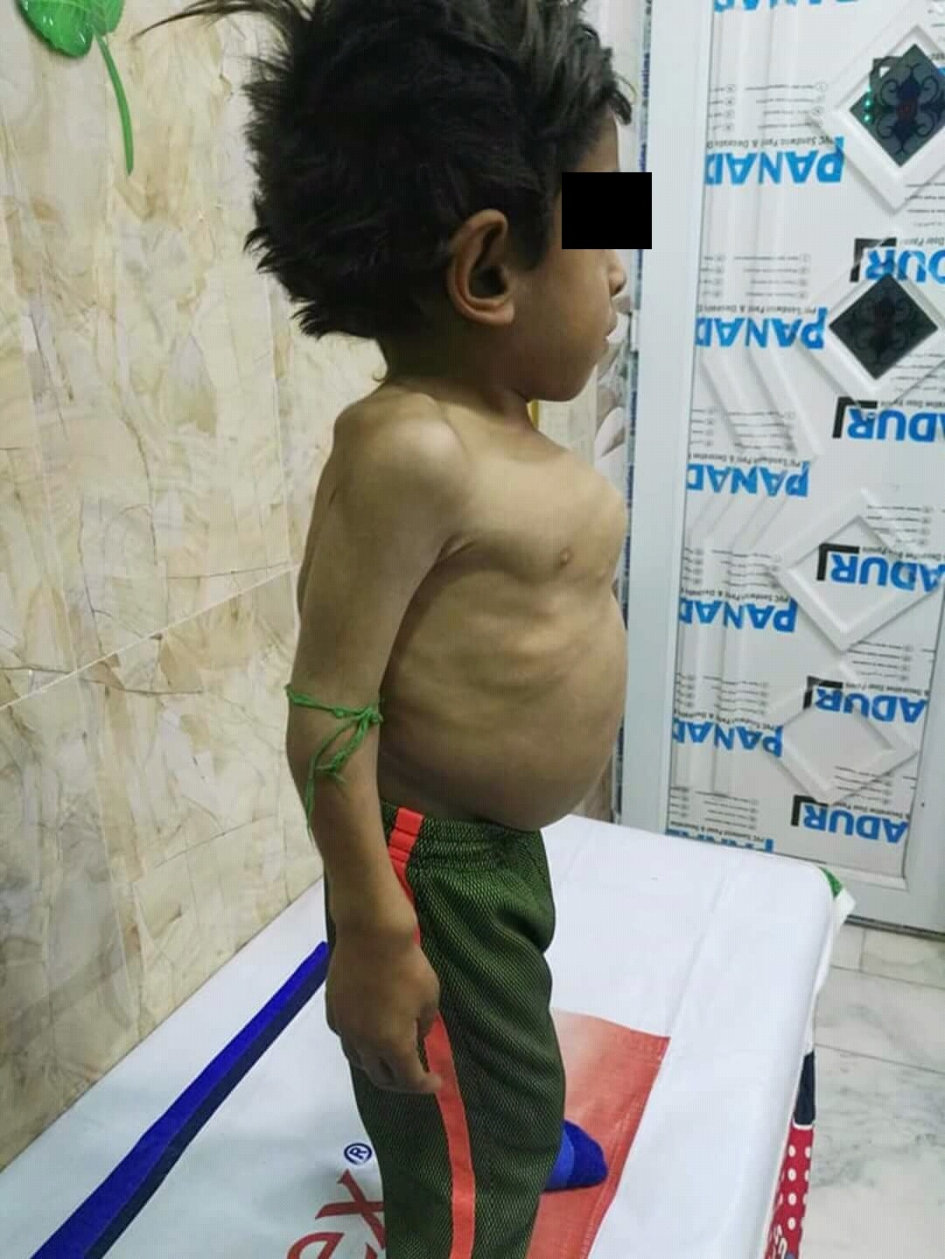
Male patient diagnosed with mucopolysaccharidosis type IV, having pigeon chest deformity and abdominal distension.

**Figure 2:**
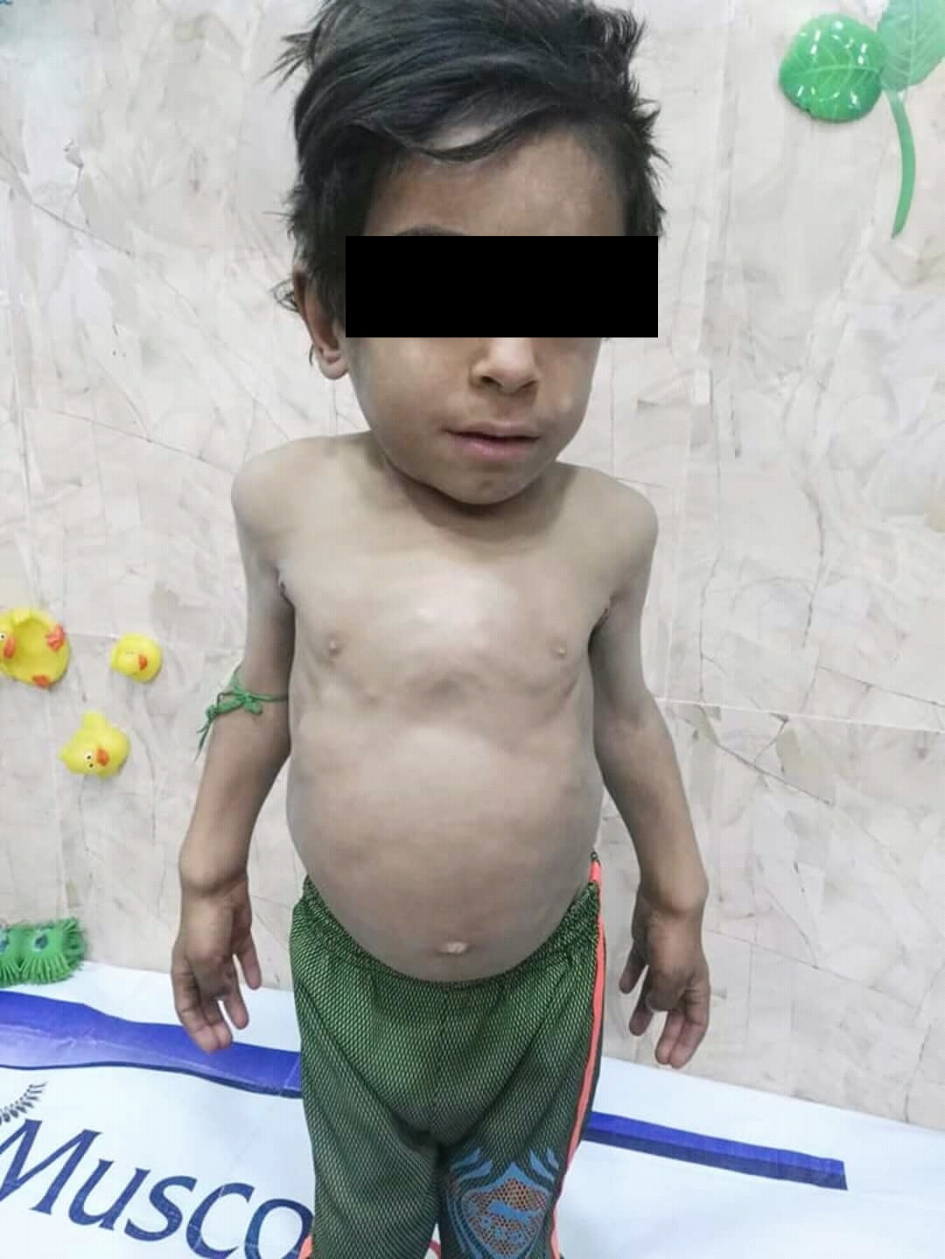
Abdominal distension in a male patient diagnosed with mucopolysaccharidosis type IV.

**Figure 3:**
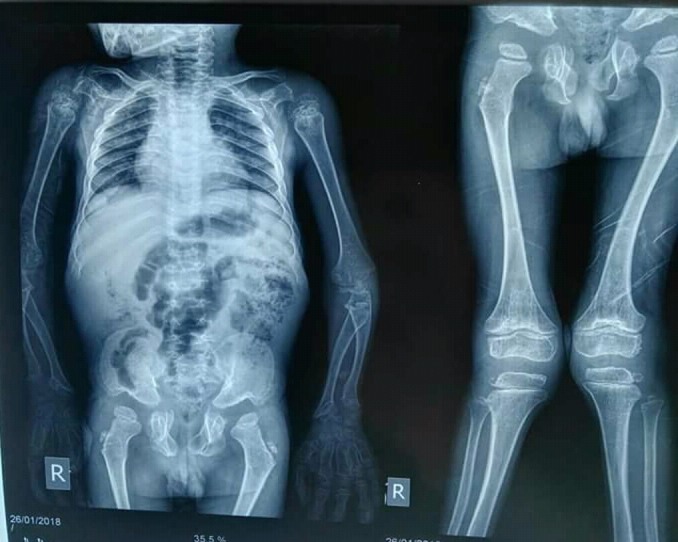
X-ray showed genu valgum in lower extremities with bowing of lower legs. No scoliosis was observed.

A seven-year-old male was brought to the clinic with an initial complaint of dwarfism and skeletal deformities. He was born from a non-consanguineous union. No family history of a similar condition was observed on either the maternal or paternal side. Upon examination, it was discerned that he had a pigeon-chest deformity, and a visible bowing of the lower extremities (Figures 1-2). The patient was then assessed and found to be with normal intelligence. Observations made were as follows: the height was in the 50th percentile with truncal dwarfism and the abdomen was distended, but no hepatosplenomegaly was observed in the patient. X-ray showed genu valgum in the lower extremities, with the bowing of lower legs as seen in Figure 3.

Further laboratory reports confirmed the presence of rickets and inclusion bodies in the lymphocytes of the patient. Due to a lack of proper infrastructure, enzyme and urine analysis were not available to confirm the enzyme defect. No cardiac or neurological abnormalities were noted on imaging. A slit-lamp examination did not reveal any corneal opacity. Following all the observations, a clinical diagnosis of Morquio syndrome was established. Initial differential diagnosis included Legg-Calve-Perthes disease, spondyloepiphyseal dysplasia, Hurler syndrome, and Hunter syndrome, among others.

The child was then referred to a geneticist, and the recommended treatment was Vimizim (BioMarin Pharmaceuticals Inc., Novato, California) (Elosulfase Alfa). Symptomatic care, and orthopedic follow-ups were also recommended for the musculoskeletal anomalies that may develop during his growth. 

## Discussion

Morquio syndrome is a rare condition, and is classified as type IV A and type IV B. An enzyme analysis will help to identify and confirm the diagnosis of Morquio syndrome in this patient. However, given the clinical presentation, the presence of inclusion bodies in the lymphocytes, and glycoaminoglycans (GAG) fragments in urine, a strong clinical diagnosis of Morquio syndrome can be made.

Treatment of Morquio syndrome includes enzyme replacement therapy of the deficient enzyme [4]. The cost of ERT is estimated to be around $380,000 per year, making it a very expensive treatment option. Hematopoietic stem cell transplantation has also been established as a treatment option for patients with Morquio syndrome [5]. Toietta G et al. performed a gene therapy for Morquio syndrome in 2004, and there are multiple similar research studies investigating cost-effective options [6].

The treatment plan for our patient that was recommended by the geneticist included possible enzyme replacement therapy. Elosulfase alfa, which goes by the tradename of Vimizim, is effective in young patients with Morquio syndrome. Some studies have found that it provides an improvement in respiratory symptoms, activities of daily living, and growth in Morquio syndrome patients [7]. Gene therapy and hematopoietic stem cell transplantation are, however, not possible given the geographic and financial settings of our patient.

## Conclusions

There is a big financial dilemma surrounding Morquio syndrome. Proper diagnosis requires an enzyme study, which is not available in many underdeveloped countries. The treatment plan for our patient included enzyme replacement therapy, which might however be very costly and a major financial burden. With newer breakthroughs in science and medicine, hopefully one day there will be a cheaper and better alternative for patients suffering from Morquio syndrome.
